# 

**Published:** 1890-09-13

**Authors:** 


SATURDAY, SEPTEMBER 13th, 1890.

**The Two Knowledges** - 353

**Words of Consolation.**—"Come" - 354

**Work, Play, and Success.** By A. Pearce Gould, M.S., F.R.C.S. - 355

**The History and Capabilities of Herbal Simples.**—By W. T. Fernie, M.D.—XVII.—Chickweed and Groundsel 356

**The Gateways of Medicines** - 357

**Everybody's Page** - 358

**The Doctor's Pulpit.**—"Plucked!" - 359

**Annotations.**—Miss Layard Turns the Tables—The late Dr. Mathews Duncan—German and English Women - 361

**Notes and News** - 360

**The Practitioner's Mirror and Retrospect:**—

Medicine—An American Medical Journal—Tropical Diarrhœa, 363;—Sunflowers as an Antiperiodic - 364

Surgery—Excision of the Mamma under Hypnotic Anæsthesia—Reparation of Bone after Trephining - 364

Therapeutics—Recent Therapentics - 365

**Short Trips for Short Holidays.**—I.—The Norman Archipelago - 367

**Passing Topics.**—The B.M.A. and the B.N.A. - 368

**General Charities** - 368

### EXTRA SUPPLEMENT—THE NURSING MIRROR.

**En Passant.**—Promptitude—The Old Style—Women's Work—An Appeal to Nurses—Asylum Attendants - xcix
**Sketches of Insanity.** By Francis H. Walmsley, M.D. X.—Epileptic Insanity, I. - c
**A Turn of the Wheel** - ci
**Everybody's Opinion.**—"Trained Midwives"—Hospital Nurses - cii
**Notes and Queries** - cii
**Vacancies** - cii
**Amusements and Relaxation** - cii

